# Activin A inhibits BMP-signaling by binding ACVR2A and ACVR2B

**DOI:** 10.1186/s12964-015-0104-z

**Published:** 2015-06-06

**Authors:** Oddrun Elise Olsen, Karin Fahl Wader, Hanne Hella, Anne Kærsgaard Mylin, Ingemar Turesson, Ingerid Nesthus, Anders Waage, Anders Sundan, Toril Holien

**Affiliations:** K.G. Jebsen Center for Myeloma Research, Department of Cancer Research and Molecular Medicine, Norwegian University of Science and Technology, Post box 8905, MTFS, N-7491 Trondheim, Norway; Departments of Oncology, and Hematology, St. Olav’s University Hospital, Trondheim, Norway; Department of Haematology, Rigshospitalet, University of Copenhagen, Copenhagen, Denmark; Department of Hematology and Coagulation Disorders, Skane University Hospital, Malmö, Sweden; Department of Medicine, Haukeland University Hospital, Bergen, Norway; Departments of Hematology, St. Olav’s University Hospital, Trondheim, Norway; CEMIR (Centre of Molecular Inflammation Research), Department of Cancer Research and Molecular Medicine, Norwegian University of Science and Technology, Trondheim, Norway

**Keywords:** Activin A, Follistatin, Receptor, BMP, Bone morphogenetic proteins, Myeloma

## Abstract

**Background:**

Activins are members of the TGF-β family of ligands that have multiple biological functions in embryonic stem cells as well as in differentiated tissue. Serum levels of activin A were found to be elevated in pathological conditions such as cachexia, osteoporosis and cancer. Signaling by activin A through canonical ALK4-ACVR2 receptor complexes activates the transcription factors SMAD2 and SMAD3. Activin A has a strong affinity to type 2 receptors, a feature that they share with some of the bone morphogenetic proteins (BMPs). Activin A is also elevated in myeloma patients with advanced disease and is involved in myeloma bone disease.

**Results:**

In this study we investigated effects of activin A binding to receptors that are shared with BMPs using myeloma cell lines with well-characterized BMP-receptor expression and responses. Activin A antagonized BMP-6 and BMP-9, but not BMP-2 and BMP-4. Activin A was able to counteract BMPs that signal through the type 2 receptors ACVR2A and ACVR2B in combination with ALK2, but not BMPs that signal through BMPR2 in combination with ALK3 and ALK6.

**Conclusions:**

We propose that one important way that activin A regulates cell behavior is by antagonizing BMP-ACVR2A/ACVR2B/ALK2 signaling.

**Electronic supplementary material:**

The online version of this article (doi:10.1186/s12964-015-0104-z) contains supplementary material, which is available to authorized users.

## Lay abstract

Activin A and bone morphogenetic proteins (BMPs) belong to a large group of signaling molecules denoted the transforming growth factor (TGF)-β superfamily. The ligands in this family bind to different complexes of type 1 and type 2 receptors, and initiate signaling through intracellular SMAD proteins. Usually, activin A and BMPs signal through SMAD2/3 and SMAD1/5/8, respectively. Activation of one type of SMAD protein may result in completely different outcome in cells compared with activation of the other type of SMAD protein.

Activin A binds strongly to the type 2 receptors ACVR2A and ACVR2B that they share with some of the BMPs. Using myeloma cell lines with well-characterized BMP-receptor expression and responses, we found that activin A inhibited signaling by BMP-6 and BMP-9 by competing for type 2 receptors. BMP-2 and BMP-4 prefer another type 2 receptor, named BMPR2, and were not inhibited by activin A. Thus, one ligand in the TGF-β superfamily may inhibit signaling by another ligand, indicating that the relative abundance of ligands determine outcome of signaling.

## Background

Members of the transforming growth factor (TGF)-β superfamily are involved in regulating diverse biological processes, including apoptosis, proliferation, organ development and bone formation. The ligands are divided into subgroups based upon their activation of different SMAD proteins and include TGF-β, bone morphogenetic proteins (BMPs), growth differentiation factors (GDFs), activins and inhibins. Ligands of the TGF-β family signal through type 1 and type 2 receptors that are conserved single transmembrane serine/threonine kinase receptors. These receptors dimerize upon ligand binding and the specificity of the ligand is commonly determined by the binding to the type 2 receptor. Thereafter the appropriate type 1 receptor is recruited. The formation of a ligand-receptor complex enables phosphorylation of the type 1 receptor which initiates downstream signaling via intracellular SMAD proteins. In most cases, TGF-β and activins signal through SMAD2/3, whereas BMPs signal through SMAD1/5/8, however this is determined by which of the type 1 receptors that is present in the ligand-bound signaling complex. For example TGF-β can activate both SMAD2/3 and SMAD1/5/8 after binding heteromeric complexes that contain both ALK5 and ALK1 type 1 receptors [1].

Activin A regulates multiple biological functions such as hormonal homeostasis, inflammation, and bone homeostasis by stimulating osteoclastogenesis and inhibiting the formation of osteoblasts. Activin A is produced by many cells in the immune system, including osteoblasts as well as by CD14^+^ osteoclast precursors [2,3]. Previously it has been shown that Activin A has an impact on macrophage polarization and may function either as a pro- or as an anti-inflammatory mediator [4]. In patients suffering from multiple myeloma activin A was upregulated in patients with advanced disease and the levels correlated with presence of bone disease [5,6]. Activin A primarily binds to the type 1 receptors ALK4 or ALK7 in complex with ACVR2A or ACVR2B, causing activation of SMAD2 or SMAD3 [7]. However, there are reports of activin A binding to and signaling through ALK2 and BMPR2 [8,9]. Follistatin is a natural antagonist of activin A which prevents activin A from interacting with its receptors [10,11]. Follistatin is also produced by osteoblasts along with activin A and the ratio between activin A and follistatin was decreased in mineralizing cultures compared to control cultures, indicating the existence of a negative feedback mechanism [2].

Using myeloma cell lines as a model system, we show that activin A antagonizes BMP-6 or BMP-9-signaling through ACVR2A/ACVR2B/ALK2, but not BMP-2 or BMP-4-signaling through BMPR2/ALK3 or BMPR2/ALK6.

## Results

**Figure 1:**
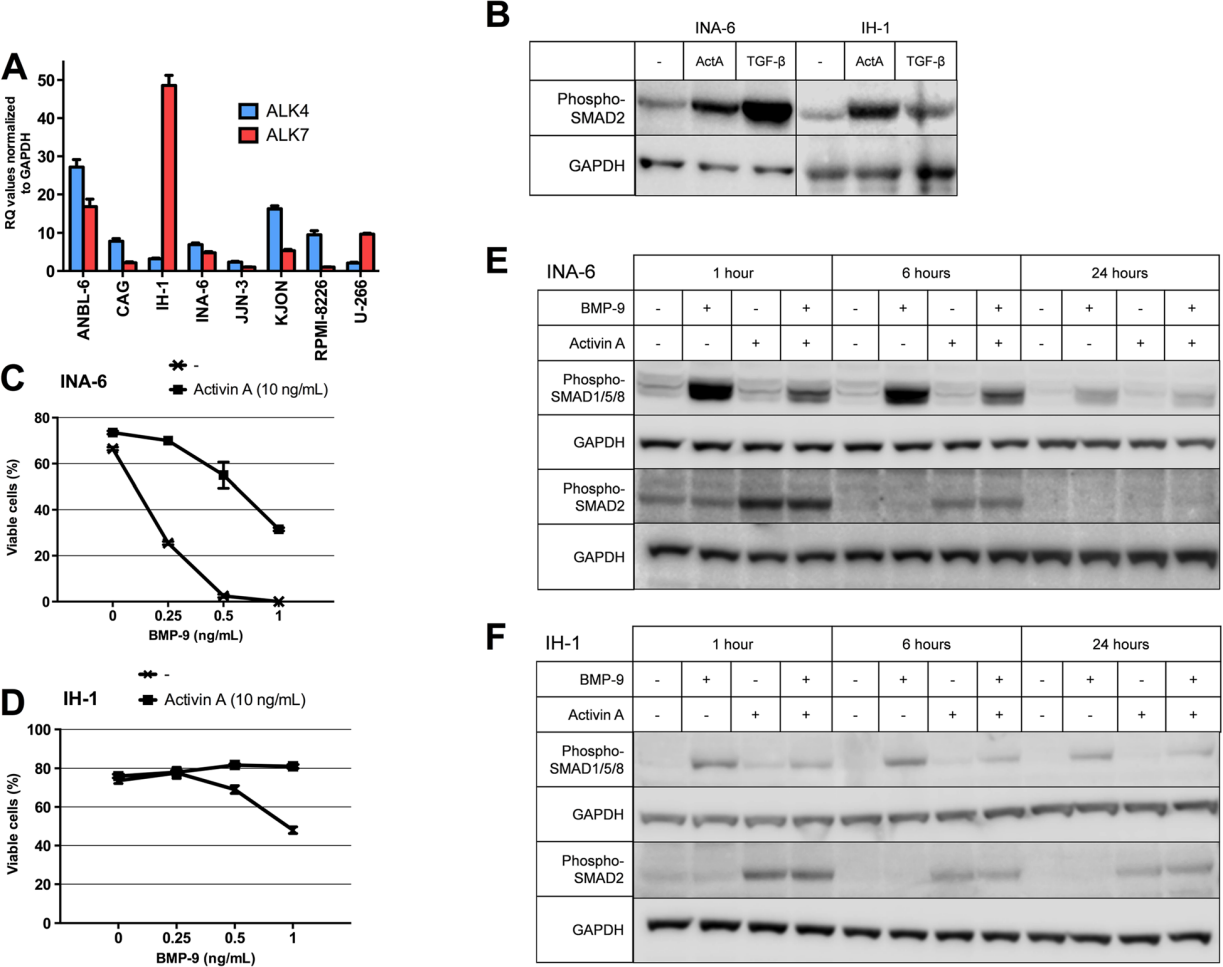
Activin A inhibited BMP-9-induced apoptosis. **(A)** The expression of the type I receptors ALK4 and ALK7 in eight human myeloma cell lines was determined using QRT-PCR. The delta delta Ct method using GAPDH as housekeeping gene was used to determine the relative levels of mRNA compared to the expression of ALK7 in cell line JJN-3 (Ct-value = 33) which was set to 1. **(B)** Phosphorylation of SMAD2 was determined using immunoblotting in INA-6 and IH-1 cells treated with activin A (10 ng/mL) or TGF-β (5 ng/mL) for 4 hours. GAPDH was used as loading control. INA-6 **(C)** and IH-1 cells **(D)** were treated for three days with activin A (10 ng/mL) and the indicated concentrations of BMP-9 before cell viability was determined by flow cytometry using annexin V/PI labeling. Cells that were negative for both annexin V and PI were considered viable. Phosphorylation of SMAD1/5/8 or SMAD2 was determined using immunoblotting in INA-6 **(E)** and IH-1 cells **(F)** treated with activin A (10 ng/mL) and/or BMP-9 (0.5 ng/mL) for one, six and 24 hours. GAPDH was used as loading control.

We have previously shown that BMPs mediated growth arrest and apoptosis in myeloma cells by activation of SMAD1/5/8 and consequent downregulation of MYC [12]. We wanted to study the impact of activin A on SMAD1/5/8-signaling and, hence, on myeloma cell growth and survival. First we measured the mRNA expression of the type 1 activin A receptors ALK4 and ALK7 in eight different myeloma cell lines. All the tested cell lines expressed both receptors albeit to varying degrees, implying that canonical activin A signaling could take place in these cells (Figure 1A). However, using CellTiter-Glo, which is a sensitive assay for cell proliferation, activin A did not influence the growth rate of any of the myeloma cell lines even after three days of treatment with activin A (data not shown). Nevertheless, treatment with either TGF-β or activin A led to phosphorylation of SMAD2, as shown here for the IH-1 and INA-6 myeloma cell lines (Figure 1B). Thus, canonical activin A- and TGF-β-signaling can take place in myeloma cells. In contrast to activin A, BMP-9 induced apoptosis in INA-6 and IH-1 cells as previously shown (Figure 1C-D) [13]. Interestingly, the effect of BMP-9 on cell viability was blunted in the presence of activin A (Figure 1C-D). BMP-induced apoptosis in myeloma cell lines is dependent on SMAD1/5/8-phosphorylation [12]. Experiments were thus performed to investigate whether activin A also prevented BMP-induced phosphorylation of SMAD1/5/8. Indeed, activin A treatment lead to inhibition of BMP-9-induced SMAD1/5/8-phosphorylation in both INA-6 and IH-1 cells (Figure 1E-F). INA-6 and IH-1 are both IL-6-dependent cell lines. To rule out that the effects of activin A were directly related to IL-6-dependency, we also looked at an IL-6-independent cell line, JJN-3. As expected, in this cell line activin A also inhibited BMP-6- and BMP-9-induced phosphorylation of SMAD1/5/8 (Additional file 1: Figure S1).

**Figure 2:**
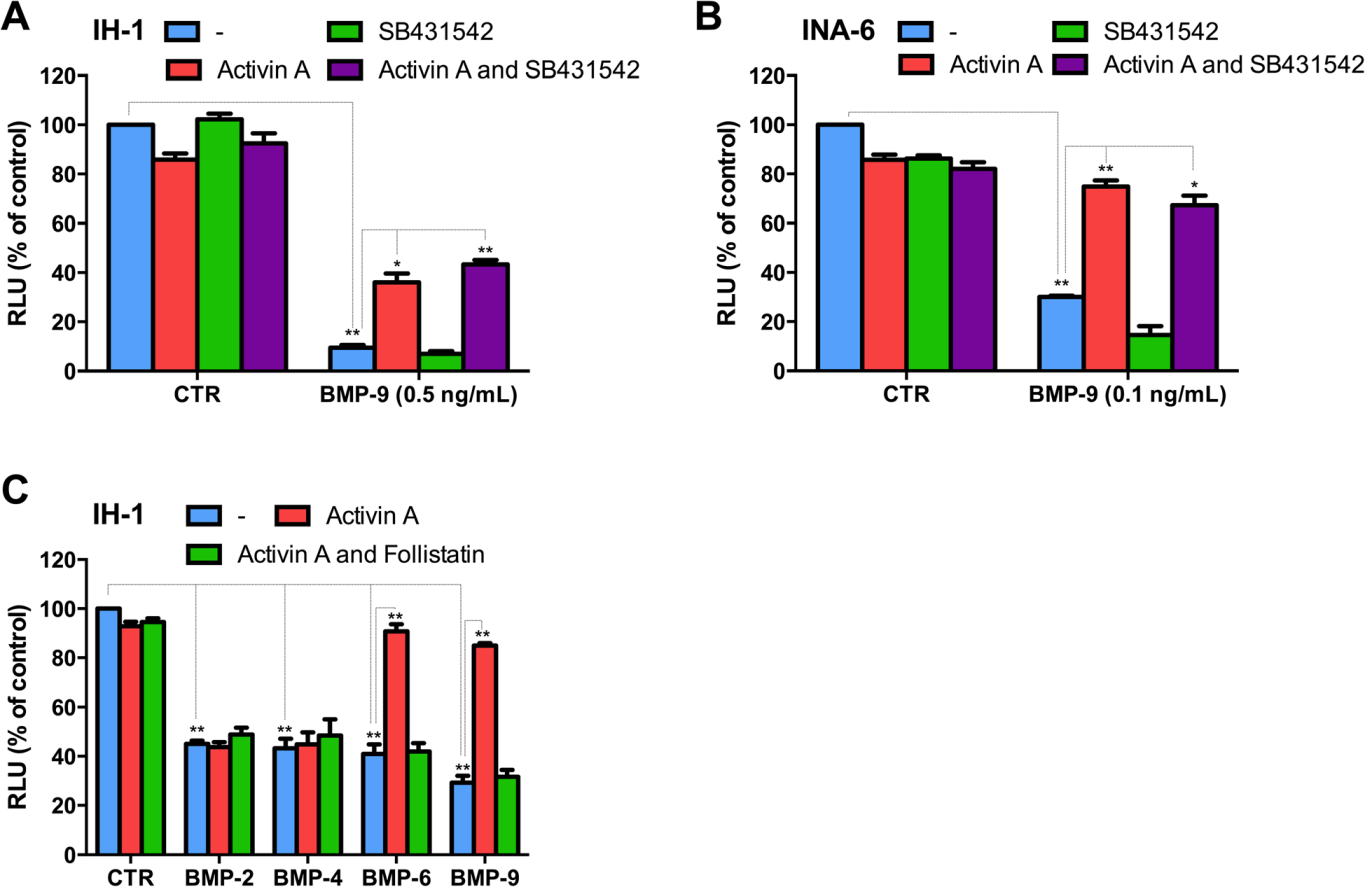
Activin A inhibited cell death induced by BMP-6 and BMP-9, but not by BMP-2 and BMP-4. IH-1 **(A)** and INA-6 cells **(B)** were treated with the indicated concentrations of BMP-9, activin A (10 ng/mL) and the inhibitor SB431542 (5 μM) for three days before cell growth was determined using the CellTiter Glo assay. Relative luciferase units (RLU) reflected the amount of ATP in each well, represented as mean ± SEM from three independent experiments. P-values were determined by *T*-test (*P < 0.05, **P < 0.01). **(C)** IH-1 cells were treated for three days with activin A (10 ng/mL), BMP-2 (5 ng/mL), BMP-4 (2.5 ng/mL), BMP-6 (25 ng/mL), BMP-9 (0.5 ng/mL) and follistatin (625 ng/mL) before cell growth was determined as in **(A)**.

In contrast to activin A, TGF-β did not antagonize BMP-induced effects in IH-1 cells (Additional file 2: Figure S2A). Treatment with BMP-9 in the presence of activin A or TGF-β showed that activin A, but not TGF-β, inhibited BMP-induced SMAD1/5/8-activation (Additional file 2: Figure S2B) suggesting that activation of SMAD2 was not sufficient to inhibit BMP-9. Furthermore, SB431542, an inhibitor of SMAD2/3 activation [14], was used to test if signaling through SMAD2 or SMAD3 was necessary for the activin A mediated inhibition of BMP-9. SB431542 treatment inhibited activin A- and TGF-β-induced SMAD2-phosphorylation (Additional file 2: Figure S2C), but did not influence the effect of activin A on BMP-induced growth inhibition in IH-1 or INA-6 cells (Figure 2A-B). Taken together, we concluded that activin A inhibited BMP-9 signaling in myeloma cells through a mechanism that was independent of activation of SMAD2 through ALK4 or ALK7.

IH-1 cells were further used to compare the effects of activin A on signaling by BMP-2, −4, −6 and −9 using the CellTiter-Glo assay. Interestingly, activin A inhibited both BMP-6- and BMP-9-signaling, but had little or no effect on BMP-2- or BMP-4-signaling (Figure 2C). As expected, addition of follistatin blunted the inhibitory effect by activin A on BMP-6 and BMP-9. The finding that activin A inhibited only two out of four BMPs suggested that the mechanism did not involve the inhibitory SMADs, SMAD-6 or SMAD-7, which are shared by the BMPs, but rather one or more specific receptors. We hypothesized that the antagonizing effect of activin A on BMP-6 and BMP-9 could be caused by competition for receptors that are shared by activin A and BMP-6 or BMP-9. BMP-6 and BMP-9 signals through the type 1 receptor ALK2 in myeloma cells, whereas BMP-2 and BMP-4 do not signal in cells that express ALK2, but lack ALK3 and ALK6, as is the case for the INA-6 myeloma cell line [12,13,15,16].

**Figure 3:**
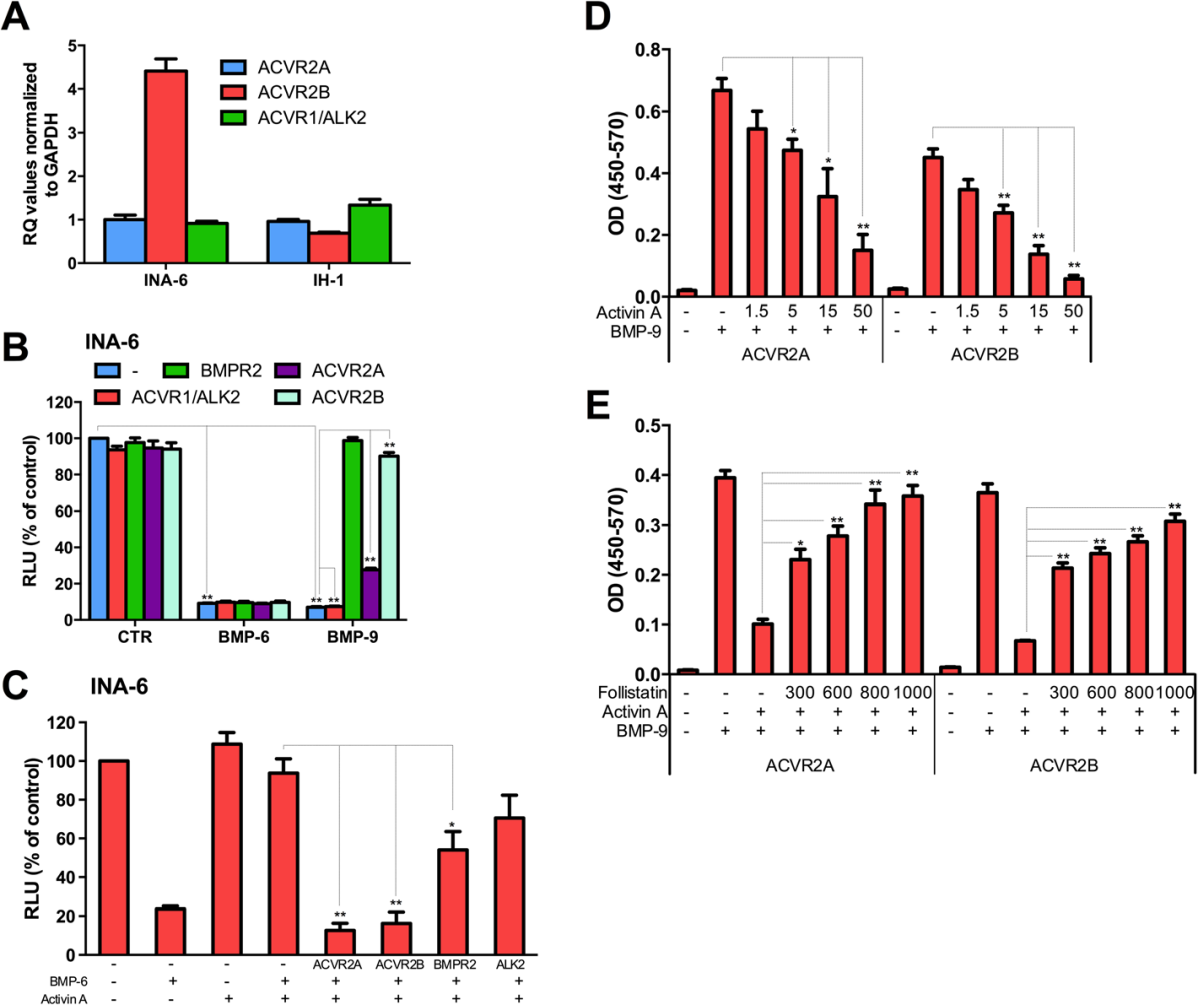
Activin A competes with BMP-6 and BMP-9 in binding to the type II receptors ACVR2A and ACVR2B. **(A)** Expression of the type II receptors ACVR2A and ACVR2B in INA-6 and IH-1 cells were determined using QRT-PCR. The delta delta Ct method using GAPDH as housekeeping gene was used to determine the relative levels of mRNA compared to the expression of ACVR2A in INA-6 (Ct-value = 33) which was set to 1. **(B)** INA-6 cells were treated with BMP-6 (50 ng/mL) or BMP-9 (0.25 ng/mL) for three days in the presence of the soluble Fc-receptors ACVR1/ALK2, BMPR2, ACVR2A or ACVR2B (5 μg/mL) and cell growth was determined using the CellTiter Glo assay. Relative luciferase units (RLU) reflected the amount of ATP in each well, represented as mean ± SEM from three independent experiments. P-values were determined by *T*-test (*P < 0.05, **P < 0.01). **(C)** INA-6 cells were treated with BMP-6 (25 ng/mL) with or without activin A (10 ng/mL) and soluble Fc-receptors (5 μg/mL) where indicated and cell growth was determined as in **(B). (D)** Soluble Fc-receptor ACVR2A and ACVR2B (1 μg/mL) was coated in wells and treated with BMP-9 (15 ng/mL) and different concentrations of activin A (1.5-50 ng/mL). Bound BMP-9 was expressed as absorbance (OD) using BMP-9 DuoSet detection reagents. Shown are mean ± SEM values from three independent experiments. P-values were determined by *T*-test (*P < 0.05, **P < 0.01). **(E)** Soluble receptors were coated into plates as in **(D)** and treated with BMP-9 (15 ng/mL), activin A (15 ng/mL) and different concentrations of follistatin (300–1000 ng/mL). Binding of BMP-9 was determined as in **(D)**.

Moreover, it was shown that BMP-2 and BMP-4 preferentially use the type 2 receptor BMPR2, whereas BMP-6 and BMP-7 use ACVR2A and ACVR2B [17]. Thus, ALK2, ACVR2A and ACVR2B are candidate mediators of the antagonizing effects of activin A on BMP-6 and BMP-9 signaling in myeloma cells. We therefore mapped the expression of receptors that could be of relevance to both BMP and activin A signaling in INA-6 and IH-1 cells. Both cell lines expressed the type 1 receptor ALK2 and the type 2 receptors ACVR2A and ACVR2B (Figure 3A).

We went on to see which of these receptors could also bind activin A. By using soluble chimeric Fc-receptors we found that the effects of BMP-6 on cells were not affected by any of the candidate receptors ALK2, ACVR2A or ACVR2B, whereas BMP-9 activity was inhibited by the type 2 receptors ACVR2A and ACVR2B (Figure 3B). This was observed even though we have shown that BMP-6 and BMP-9 both signal through ALK2 in these cells [12,13]. A likely reason for this is that the affinity of either of these BMPs to ALK2 alone is weak and not enough to cause binding. Since BMP-6 was not affected by any of the soluble Fc-receptors, we could use this assay to study the effects of one receptor at a time with regards to activin A binding. We hypothesized that activin A could bind stronger than BMP-6 to ALK2, ACVR2A or ACVR2B. Indeed, we found that activin A’s inhibitory effect against BMP-6 was prevented by the addition of soluble ACVR2A or ACVR2B (Figure 3C), indicating that activin A could compete with BMP-6 for binding to ACVR2A and ACVR2B. To directly show that activin A competed with BMP-9 for binding to ACVR2A and ACVR2B, we measured the binding of BMP-9 in the absence and presence of activin A to recombinant receptors in a cell-free assay. The presence of activin A inhibited BMP-9 binding to isolated receptors in a dose dependent manner (Figure 3D). Furthermore, this inhibition of BMP-9 binding by activin A could be reverted by adding follistatin (Figure 3E). The reduction of 15 ng/mL BMP-9 binding to about 50% by addition of 15 ng/mL activin A suggests that BMP-9 and activin A have approximately similar affinities for these receptors.

## Discussion

BMPs are extremely potent in inducing myeloma cell death and thus, myeloma cells must have ways to escape the tumor suppressing effects of BMPs. The main finding reported here is that activin A, by sharing receptors with BMP-6 and BMP-9, but not BMP-2 and BMP-4, may inhibit BMP-6 and BMP-9-induced apoptosis in myeloma cells. In myeloma cells, BMP-6- and BMP-9-induced activation of SMAD1/5/8 through ACVR2A/ACVR2B/ALK2 was inhibited by activin A treatment. Furthermore, we showed directly that activin A and BMP-9 competed for binding to isolated recombinant ACVR2A and ACVR2B.

The activin- and BMP-receptors are shared by multiple ligands. Moreover, ACVR2A, ACVR2B and ALK2 are ubiquitously expressed and knowledge about regulation of signaling through these receptors might have general implications, for instance in bone homeostasis in myeloma patients. It has been shown that elevated levels of activin A were associated with poor prognosis and severity of bone disease [5,6]. Moreover, activin A inhibited SMAD1/5/8 activity, but the role of different BMPs in bone homeostasis is still unclear. Thus, it is currently not known which BMP is the most important for bone formation *in vivo* and to what extent inhibition of BMP activities plays a role for formation of osteolytic lesions in myeloma patients. It could be hypothesized that BMP-6 and BMP-9 which both are affected by activin A are important for bone homeostasis in myeloma patients. Further experiments are needed to address this issue.

Unlike most BMPs, activin A has been shown to have a strong affinity for type 2 receptors. Thus, when decoy receptors for ACVR2A or ACVR2B were used in clinical trials, activin A signaling was inhibited with improved anemia and bone disease [18,19]. It is therefore tempting to speculate that the effects on anemia and bone disease also could be due to increased BMP-6 or BMP-9 activity, due to activin A inhibition.

The repertoire of type 1 and type 2 receptors, as well as type 3 receptors and other co-factors expressed on a given cell determine the effects of a given TGF-β superfamily ligand. It is still unclear which combination of type 1 and type 2 receptors are used by the different BMPs. It could be redundancy in the system, so that any one of the type 2 receptors could be used by many of the BMPs, or there may be a specific type 2 receptor needed for a specific combination of BMP and type 1 receptor. These are unresolved questions and the answers to these would provide more insight into how to regulate activin A activity in a more controlled and specific manner.

Another determining factor for activin A signaling is the presence of natural antagonists. The best known antagonist of activin A is follistatin which binds to activin A with high affinity and also may cause its degradation [10,11]. Thus, the ratio between follistatin and activin A should determine activin A’s ability to inhibit BMP-6 or BMP-9, both with regards to myeloma cell apoptosis and to osteoblastogenesis.

## Conclusions

The main finding presented here is that activin A regulated cell behavior by antagonizing BMPs that signal through ACVR2A/ACVR2B/ALK2. Our results provide further knowledge on the mechanisms behind activin A function on the cellular level. Due to an increasing number of clinical trials using different inhibitors of activin A function, this information may be useful to understand and to avoid possible side effects.

## Methods

### Cell lines and reagents

The human multiple myeloma cell lines INA-6, ANBL-6, CAG and JJN-3 were kind gifts from Dr. M. Gramatzki (University of Erlangen-Nurnberg, Erlangen, Germany), Dr. D. Jelinek (Mayo Clinic, Rochester, MN, USA), Dr. J. Epstein (University of Arkansas for Medical Sciences, Little Rock, AR, USA) and Dr. J. Ball (University of Birmingham, UK), respectively. RPMI-8226 and U266 were from American Type Culture Collection (Rockville, MD, USA). IH-1 [20] and KJON were established in our laboratory. INA-6 and ANBL-6 cells were grown in 10% heat inactivated fetal calf serum (FCS) in RPMI-1640 (RPMI) supplemented with recombinant human interleukin (IL)-6 (1 ng/mL). IH-1 and KJON cells were maintained in 10% (IH-1) or 5% (KJON) heat inactivated human serum (HS) (Department of Immunology and Transfusion Medicine, St. Olav’s University Hospital, Trondheim, Norway) in RPMI and IL-6 (2 ng/mL). CAG, JJN-3, RPMI-8226 and U266 were grown in RPMI supplemented with 10, 10, 20 or 15% FCS, respectively. Cells were cultured at 37°C in a humidified atmosphere containing 5% CO_2_. For experiments 2% HS in RPMI was used, with IL-6 (1 ng/mL) added for IL-6 dependent cells. All recombinant human proteins (Activin A, follistatin, TGF-β, BMP-2, BMP-4, BMP-6, BMP-9, ALK2-Fc, BMPR2-Fc, ACVR2A-Fc, ACVR2B-Fc) were from R&D Systems (R&D Systems Europe Ltd., Abingdon, UK), except IL-6 (Biosource, Camarillo, CA, USA) and SB431542 (Sigma-Aldrich, St Louis, MO, USA).

### Cell viability

To measure changes in cell viability, cells were stained using the Apotest FITC kit (Nexins Research, Kattendijke, the Netherlands). The cells were incubated with annexin V FITC (0.2 μg/mL in annexin binding buffer) for 1 h on ice. Propidium iodide (PI) (1.4 μg/mL) was added 5 min prior to data acquisition using an LSRII flow cytometer (BD Biosciences). Cells negative for both annexin V and PI staining were considered viable.

### Cell proliferation

CellTiter-Glo (Promega, Madison, WI, USA) measures ATP-levels in cells by use of luciferase and was used to determine relative cell proliferation. Cells were seeded in 96-well optical plates and treated as indicated. CellTiter-Glo reagent was added according to the manufacturer’s protocol and luminescence was determined using Victor 1420 multilabel counter (PerkinElmer Inc., Waltham, MA, USA).

### Western blotting

Cells were treated as indicated, washed with ice cold phosphate-buffered saline (PBS) and lysed for 30 minutes on ice. The lysis buffer contained 1% Nonidet P40 (NP-40) (Sigma-Aldrich), 150 mM NaCl, 50 mM Tris–HCl (pH 7.5), protease inhibitor cocktail (Roche, Basel, Switzerland), 1 mM Na_3_VO_4_ and 50 mM NaF. Samples were separated on NuPAGE Bis-Tris gels with MOPS running buffer (Invitrogen, Carlsbad, CA, USA). Gels were blotted onto nitrocellulose membranes, blocked with 5% nonfat dry milk in Tris-buffered saline with 0.01% Tween 20 (TBS-T) and incubated over night with primary antibodies as indicated. Primary antibodies used were: phospho-SMAD1/5/8 (RRID:AB_331672, Cat# 9511 L, Cell Signaling Technology, Beverly, MA, USA), phospho-SMAD2 (RRID:AB_1587251, Cat# 04–953, Millipore A/S, Oslo, Norway) and GAPDH (RRID:AB_2107448, Cat# Ab8245, Abcam, Cambridge, UK). Blots were washed in TBS-T before incubation for one hour with horseradish peroxidase conjugated secondary antibodies (Dako Cytomation, Glostrup, Denmark). The blots were washed thoroughly with TBS-T before bands were detected using SuperSignal West Femto (Thermo Fisher Scientific, Waltham, MA, USA) as luminescence substrate and Licor Odyssey FC (LI-COR Biosciences, NE, USA).

### QRT-PCR

Total RNA was isolated using the High Pure RNA Isolation Kit (Roche Applied Science, Mannheim, Germany), and complementary DNA (cDNA) was synthesized using the High Capacity RNA-to-cDNA kit (Applied Biosystems, Foster City, CA, USA). PCR was performed using StepOne real-time PCR System and Taqman Gene Expression Assays (Applied Biosystems). The Taqman assays used were: ACVR1B/ALK4 (Hs00244715_m1), ACVR1C/ALK7 (Hs00899854_m1), ACVR1/ALK2 (Hs00153836_m1), ACVR2A (Hs00155658_m1), ACVR2B (Hs00609603_m1), and GAPDH (Hs99999905_m1). The comparative Ct method was used to estimate relative changes in receptor expression using GAPDH as housekeeping gene.

### ELISA

Ninety-six-well MaxiSorp plates were coated over night at 4°C with 1 μg/mL of the receptors ACVR2A and ACVR2B in PBS. The wells were blocked for one hour at room temperature with 1% BSA (R&D Systems) in PBS before addition of BMP-9, activin A, follistatin or combinations of these. Bound BMP-9 was detected using detection reagents from BMP-9 DuoSet ELISA (R&D Systems), and optical density was determined using iMark™ Microplate Absorbance Reader (Bio-Rad, Hercules, CA, USA).

### Statistics

Statistical calculations were done using GraphPad Prism 6 (GraphPad Software, Inc., La Jolla, CA, USA). Significance was analyzed using two-sided, unpaired *T*-test with Welch’s correction. Differences of P < 0.05 or **P < 0.01 were considered significant.

## Additional files

Additional file 1:
**Olsen OE** ***et al.*** **Activin A inhibits BMP-signaling by binding ACVR2A and ACVR2B.** The effect of activin A is also seen in the JJN-3 IL-6 independent cell line. JJN-3 cells were treated for one hour with BMP-6 (100 ng/mL) or BMP-9 (5 ng/mL) with or without activin A (10 ng/mL) before phosphorylation of SMAD1/5/8 and SMAD2 was determined using immunoblotting. GAPDH was used as loading control. Additional file 2:
**Olsen OE** ***et al.*** **Activin A inhibits** **BMP-signaling** **by binding ACVR2A and ACVR2B. TGF-β does not inhibit BMP-6 or BMP-9 in myeloma cells.** (A) IH-1 cells were treated for three days with BMP-2 (5 ng/mL), BMP-4 (2.5 ng/mL), BMP-6 (25 ng/mL) and BMP-9 (0.5 ng/mL), and with or without TGF-β (5 ng/mL) before cell growth was determined. The CellTiter Glo assay was used and relative luciferase units (RLU) reflected the amount of ATP in each well. Error bars represent +/−1 SD of three technical replicates. (B) Immunoblotting was used to determine phosphorylation of SMAD1/5/8 and SMAD2 in IH-1 cells treated for 4 hours with activin A (10 ng/ml) or TGF-β (5 ng/mL) with or without BMP-6 (25 ng/mL) and BMP-9 (0.5 ng/mL). GAPDH was used as loading control. (C) Effect of the inhibitor SB431542 (5 μM) was shown with immunoblotting of IH-1 cells treated with activin A (10 ng/ml) or TGF-β (5 ng/mL).
