# Supplementary material for: Activin A inhibits BMP-signaling by binding ACVR2A and ACVR2B
Source: Cell Commun Signal. 2015 Jun 6;13:27. doi: 10.1186/s12964-015-0104-z (PMC4467681; doi:10.1186/s12964-015-0104-z)
Supplement: Additional file 1: — Olsen OE et al. Activin A inhibits BMP-signaling by binding ACVR2A and ACVR2B. The effect of activin A is also seen in the JJN-3 IL-6 independent cell line. JJN-3 cells were treated for one hour with BMP-6 (100 ng/mL) or BMP-9 (5 ng/mL) with or without activin A (10 ng/mL) before phosphorylation of SMAD1/5/8 and SMAD2 was determined using immunoblotting. GAPDH was used as loading control. [file 12964_2015_104_MOESM1_ESM.pdf]

Additional file 1

Olsen OE *et al.* Activin A inhibits BMP-signaling by binding ACVR2A and ACVR2B

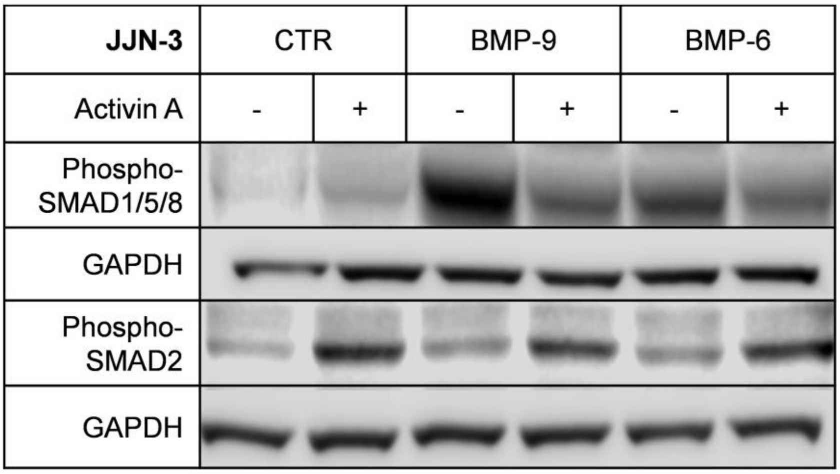

Additional Figure 1. The effect of activin A is also seen in the JJN-3 IL-6 independent cell line

JJN-3 cells were treated for one hour with BMP-6 (100 ng/mL) or BMP-9 (5 ng/mL) with or without activin A (10 ng/mL) before phosphorylation of SMAD1/5/8 and SMAD2 was determined using immunoblotting. GAPDH was used as loading control.
