# Supplementary material for: Activin A inhibits BMP-signaling by binding ACVR2A and ACVR2B
Source: Cell Commun Signal. 2015 Jun 6;13:27. doi: 10.1186/s12964-015-0104-z (PMC4467681; doi:10.1186/s12964-015-0104-z)
Supplement: Additional file 2: — Olsen OE et al. Activin A inhibits BMP-signaling by binding ACVR2A and ACVR2B. TGF-β does not inhibit BMP-6 or BMP-9 in myeloma cells. (A) IH-1 cells were treated for three days with BMP-2 (5 ng/mL), BMP-4 (2.5 ng/mL), BMP-6 (25 ng/mL) and BMP-9 (0.5 ng/mL), and with or without TGF-β (5 ng/mL) before cell growth was determined. The CellTiter Glo assay was used and relative luciferase units (RLU) reflected the amount of ATP in each well. Error bars represent +/−1 SD of three technical replicates. (B) Immunoblotting was used to determine phosphorylation of SMAD1/5/8 and SMAD2 in IH-1 cells treated for 4 hours with activin A (10 ng/ml) or TGF-β (5 ng/mL) with or without BMP-6 (25 ng/mL) and BMP-9 (0.5 ng/mL). GAPDH was used as loading control. (C) Effect of the inhibitor SB431542 (5 μM) was shown with immunoblotting of IH-1 cells treated with activin A (10 ng/ml) or TGF-β (5 ng/mL). [file 12964_2015_104_MOESM2_ESM.pdf]

Additional file 2

Olsen OE *et al.* Activin A inhibits BMP-signaling by binding ACVR2A and ACVR2B

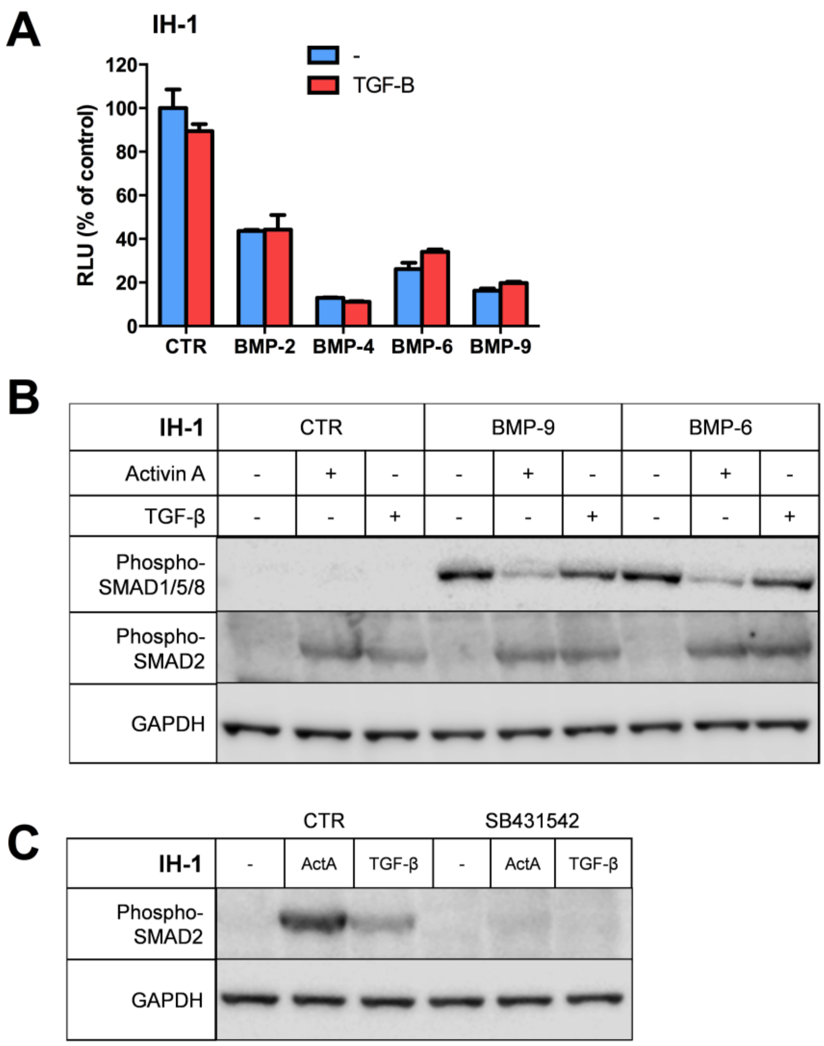

Additional Figure 2. TGF-β does not inhibit BMP-6 or BMP-9 in myeloma cells

(A) IH-1 cells were treated for three days with BMP-2 (5 ng/mL), BMP-4 (2.5 ng/mL), BMP-6 (25 ng/mL) and BMP-9 (0.5 ng/mL), and with or without TGF-β (5 ng/mL) before cell growth was determined. The CellTiter Glo assay was used and relative luciferase units (RLU) reflected the amount of ATP in each well. Error bars represent +/-1 SD of three technical replicates. (B) Immunoblotting was used to determine phosphorylation of SMAD1/5/8 and SMAD2 in IH-1 cells treated for 4 hours with activin A (10 ng/ml) or TGF-β (5 ng/mL) with or without BMP-6 (25 ng/mL) and BMP-9 (0.5 ng/mL). GAPDH was used as loading control. (C) Effect of the inhibitor SB431542 (5 µM) was shown with immunoblotting of IH-1 cells treated with activin A (10 ng/ml) or TGF-β (5 ng/mL).
